# Assessing the Impact of Misclassification Error on an Epidemiological Association between Two Helminthic Infections

**DOI:** 10.1371/journal.pntd.0000995

**Published:** 2011-03-29

**Authors:** Mushfiqur R. Tarafder, Hélène Carabin, Stephen T. McGarvey, Lawrence Joseph, Ernesto Balolong, Remigio Olveda

**Affiliations:** 1 Department of Biostatistics and Epidemiology, College of Public Health, University of Oklahoma Health Sciences Center, Oklahoma City, Oklahoma, United States of America; 2 Department of Family, Community and Rural Health, The Commonwealth Medical College, Scranton, Pennsylvania, United States of America; 3 International Health Institute, Brown University, Providence, Rhode Island, United States of America; 4 Division of Clinical Epidemiology, McGill University Health Centre, Montréal, Canada; 5 Research Institute for Tropical Medicine, Alabang, Muntinlupa City, Philippines; George Washington University, United States of America

## Abstract

**Background:**

Polyparasitism can lead to severe disability in endemic populations. Yet, the association between soil-transmitted helminth (STH) and the cumulative incidence of *Schistosoma japonicum* infection has not been described. The aim of this work was to quantify the effect of misclassification error, which occurs when less than 100% accurate tests are used, in STH and *S. japonicum* infection status on the estimation of this association.

**Methodology/Principal Findings:**

Longitudinal data from 2276 participants in 50 villages in Samar province, Philippines treated at baseline for *S. japonicum* infection and followed for one year, served as the basis for this analysis. Participants provided 1–3 stool samples at baseline and 12 months later (2004–2005) to detect infections with STH and *S. japonicum* using the Kato-Katz technique. Variation from day-to-day in the excretion of eggs in feces introduces individual variations in the sensitivity and specificity of the Kato-Katz to detect infection. Bayesian logit models were used to take this variation into account and to investigate the impact of misclassification error on the association between these infections. Uniform priors for sensitivity and specificity of the diagnostic test to detect the three STH and *S. japonicum* were used. All results were adjusted for age, sex, occupation, and village-level clustering. Without correction for misclassification error, the odds ratios (ORs) between hookworm, *Ascaris lumbricoides*, and *Trichuris trichiura*, and *S. japonicum* infections were 1.28 (95% Bayesian credible intervals: 0.93, 1.76), 0.91 (95% BCI: 0.66, 1.26), and 1.11 (95% BCI: 0.80, 1.55), respectively, and 2.13 (95% BCI: 1.16, 4.08), 0.74 (95% BCI: 0.43, 1.25), and 1.32 (95% BCI: 0.80, 2.27), respectively, after correction for misclassification error for both exposure and outcome.

**Conclusions/Significance:**

The misclassification bias increased with decreasing test accuracy. Hookworm infection was found to be associated with increased 12-month cumulative incidence of *S. japonicum* infection after correction for misclassification error. Such important associations might be missed in analyses which do not adjust for misclassification errors.

## Author Summary

Hookworm, roundworm, and whipworm are collectively known as soil-transmitted helminths. These worms are prevalent in most of the developing countries along with another parasitic infection called schistosomiasis. The tests commonly used to detect infection with these worms are less than 100% accurate. This leads to misclassification of infection status since these tests cannot always correctly indentify infection. We conducted an epidemiological study where such a test, the Kato-Katz technique, was used. In our study we tried to show how misclassification error can influence the association between soil-transmitted helminth infection and schistosomiasis in humans. We used a statistical technique to calculate epidemiological measures of association after correcting for the inaccuracy of the test. Our results show that there is a major difference between epidemiological measures of association before and after the correction of the inaccuracy of the test. After correction of the inaccuracy of the test, soil-transmitted helminth infection was found to be associated with increased risk of acquiring schistosomiasis. This has major public health implications since effective control of one worm can lead to reduction in the occurrence of another and help to reduce the overall burden of worm infection in affected regions.

## Introduction

Polyparasitism is a common feature in parasite endemic regions, which includes most developing countries [1], [2]. High prevalence of co-infection with soil-transmitted helminths (STHs), which include roundworm (*Ascaris lumbricoides*), whipworm (*Trichuris trichiura*), and hookworm (*Ancylostoma duodenale* and *Necator americanus*), and *Schistosoma* spp. has been reported [3], [4]. Together, these infections correspond to an estimated 43.5 million disability-adjusted life years (DALYs) lost annually [5], [6]. Schistosomiasis and STH infections are associated with conditions of poverty, such as poor hygiene, lack of safe water, inadequate sanitation and factors such as water management systems, age, gender, and farming related activities [4], [5], [7]–[14].

Laboratory studies suggest that infection with one helminth may influence the outcome of infection with another helminth [15]. Positive cross-sectional correlation and synergism between schistosome and STH infections have been reported [2], [3], [6], [16]–[18]. Immunosuppressive effect of STH has been reported, particularly with hookworm infections [19], [20]. The influence of STH infection on risk of infection with schistosomes has not been epidemiologically investigated. One challenge faced by investigators is the use of a less than perfect diagnostic test. The outcome, exposures, confounding variables, or any combination of these can contain errors [21]–[23]. Error in identification of infection status occurs when the test used to identify the infection is not 100% accurate, or not a ‘gold’ standard test [21], [24], [25].

*Schistosoma japonicum* and STH infections are most commonly detected by examining a stool sample under the microscope for the presence of parasitic eggs. Variation from day-to-day in the excretion of *S. japonicum* and STH eggs in human feces has been reported [26]–[29]. Collecting stool samples over consecutive days has been shown to improve the sensitivity of coprological tests like Kato-Katz [29], [30]. However, in practice, an unequal number of stool specimens per subject are collected as it is difficult to collect the desired number of stool samples from each subject. This produces potential complications in diagnosing *S. japonicum* and STH infections as the sensitivity and specificity of the diagnostic tests vary according to the number of stool samples examined [31], [32].

The purpose of this study was to show the impact of adjusting for misclassification error in estimating the effect of STH infections on the 12-months cumulative incidence of *S. japonicum* infection. Measuring such impact will contribute to a better understanding of the association between STH and schistosomiasis.

## Materials and Methods

### Ethics statement

The research was approved by the institutional review board (IRB) of the Brown University in the United States and the IRB of the Research Institute for Tropical Medicine in the Philippines. The data analysis component of the study was reviewed and approved by the University of Oklahoma Health Sciences Center IRB. The chiefs of all villages were asked permission for the village to be included in the study. In addition, all eligible participants were asked for their consent to participate. Only those individuals who provided written informed consent were included. Written informed consent for individuals below 18 years old was obtained and provided by parents or legal guardians.

### Source of data

We used data from a longitudinal study conducted between January 2004 and December 2005 in the province of Samar, the Philippines. The main purpose of the original study was to assess the effect of water and animal management systems on the transmission of *S. japonicum* infection. The design of the baseline study was described elsewhere [33]. A brief summary is given below.

### Study population

Seventy-five out of 134 villages endemic for *S. japonicum* in Samar in 2002 were eligible for participation [33]. The inclusion criteria were safety and accessibility of the field team, location and number of households in each village. Twenty-five primarily rain-fed villages and 25 villages with some form of man-made irrigation system were selected.

Eligible households were those of at least five members and where at least one member was working full time in a rain-fed farm in “rain-fed” villages and at least 50% of the time in a man-made irrigated farm in “irrigated” villages. A maximum of 35 eligible households were randomly selected from each village using the following procedure. A list of 50 random numbers was created (one list per village). Eligible households were allocated consecutive numbers and visited in the order chosen at random. If a household refused to participate, the next available household was asked to participate. When 35 or fewer households were eligible in a village, they were all invited to participate in the study. At most six individuals including at least one full-time rice farmer were selected at random from each household.

### Baseline data and stool collection

An individual-level interview included questions on age, gender, and occupation. Participants were asked to provide one stool sample (morning or first) per day for three consecutive days. Each participant provided between one and three stool samples. If a participant provided a stool sample on one of the three days but was unable for any reason to provide stool samples on other days, that person was still considered as a stool sample provider. Stool envelopes (of wax paper and book paper) with popsicle sticks were distributed to participants a day before the actual stool collection. At least thumb-size stool samples were submitted. Portions from different parts of the stool were taken to fill up the template. Although consistency of the stool sample was not recorded, only pasty to formed stool could be accommodated in the stool envelopes. Stool samples were processed 2–3 h after collection. Two slides were prepared from each stool sample. All slides were placed in a styrofoam box with cold packs inside at the end of each collection day. At the end of each collection week all slides were brought to a designated laboratory and transferred to a refrigerator. The time delay between stool sample processing and microscopic reading associated with day one stool collection (provided by 99.45% of participants) ranged from less than 24 hours to as long as 20 days with a median of 4 days (inter-quartile range: 2–6 days). Stool samples were examined for the presence of eggs of *S. japonicum* and the three STHs. No distinction between *N. americanus* and *A. duodenale* eggs was made, although prior reports from the Philippines found exclusively *Necator* spp. infections [34]. The Kato-Katz technique was used to detect the helminth eggs in stool samples [35]. The number of eggs per gram of stool (epg) was counted for *S. japonicum*. Although the eggs of each of the STHs were originally documented qualitatively in five response categories (0, + through ++++), STHs were considered as dichotomous variables (observed infected or uninfected) since the researchers were particularly interested in this association. Also, since the infection of interest of the original study was schistosomiasis, the semi-quantitative ascertainment of STH infection may not have been as accurate as that for schistosomiasis. Laboratory technicians were blinded to the identity of the provider of the stool sample they were preparing and reading and did not know if two stool samples were from the same participant (two consecutive day's sample).

### Mass treatment

Details about the mass treatment have been published elsewhere [36]. Briefly, following the baseline data and stool collection, all residents who were ≥5 years of age at the time and living in the 50 study villages were offered praziquantel. Praziquantel was administered in two equal split doses to give each individual a total of 60 mg/kg. The split doses were administered 4 hours apart with the first dose usually between 9 am and noon. All participants who provided baseline stool samples had been notified of their test results before treatment was offered. Before mass treatment, community preparation was implemented and an effort was made to ensure all cases found to be positive for *S. japonicum* were treated. Despite these efforts, the village-level participation proportion varied from 16% to 81% [36]. The parasitological test results were shared with the local ministry of health and the national schistosomiasis control team and it was decided to treat villagers positive to STH at the end of the whole study, that is, after the 12-months follow-up. This approach was approved by both IRBs.

### Follow-up stool sample

All of the study participants were asked to provide three stool samples over three consecutive days 12 months after the mass treatment. All individuals who provided at least one stool sample were considered as follow-up stool sample providers. Stool samples were processed and examined in the same manner and by the same people as at baseline.

### Statistical analysis

Some of the participants who provided the baseline stool samples did not participate in the mass treatment program. Moreover, not all participants provided stool samples during the follow-up survey. The 12-month cumulative incidence of *S. japonicum* infection/reinfection following mass treatment can only be calculated among the “at-risk” participants who provided at least one stool sample at baseline and follow-up and received treatment. For the purpose of this study, we assumed 100% efficacy of praziquantel for the treatment of schistosomiasis.

As mentioned earlier, we obtained between one and three stool samples on consecutive days from each participant at baseline and follow-up. This introduces individual variations in the sensitivity and specificity of the Kato-Katz to detect infection. To take this variation into account, and to adjust for the village-level clustering of infection, we used a Bayesian latent class hierarchical cumulative-logit regression model based on a method described by Joseph and others (1995) and adapted to our problem (1, 2, or 3 days of sampling) for *S. japonicum* in animals and in humans in the Philippines [25], [33], [37], [38].

The probability of any single test being positive is the sum of the probability of a true positive result and the probability of a false positive result. If *P* is the total probability of a positive test, then, from the properties of diagnostic tests, we have
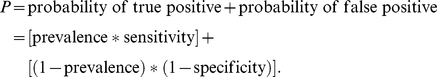



When there is more than one test per person, the properties of multiple tests can be modeled using probability *P* as the probability parameter of a binomial distribution, assuming that the tests are independent from each other [37]. In the absence of a ‘gold’ standard test, the true status of each subject is unknown, and hence can be considered as ‘latent data’. According to Bayes' theorem, the joint posterior distribution is proportional to the product of the likelihood function and prior distribution, from which all inferences can be obtained. The posterior distribution is not directly available, but inferences about each parameter are available using a Gibbs sampler algorithm, as has become standard in Bayesian analysis. The unknown true infection status for each subject can be estimated once the sensitivity and specificity have been estimated.

The main outcome of interest here is the probability distribution of the true *S. japonicum* infection category at follow-up. *S. japonicum* epg counts were grouped into three categories namely: uninfected (0 epg), light infection (1 to 100 epg) and moderate to heavy infection (over 100 epg) [33]. With a three-category outcome variable, classification errors must be further subdivided. For example, when a participant who is truly negative tests positive, there are two possible errors and 1-specificity or the false positive rate must be divided into light or moderate/heavy misclassification errors. The exposure of interest is the probability distribution of true STH infection status (for a particular STH) classified as positive or negative. Separate models were carried out for each of the three STHs.

Each hierarchical model consists of three levels, as follows: the first level includes one intercept parameter for each village and independent variables for age, sex, occupation, and one of the STHs under study. At the second level of the hierarchical model, the intercept parameters from each of the 50 villages are modeled as a linear regression to account for the clustering of infection within village. At the third level, prior distributions were specified for all parameters. Uniform (uninformative) prior distributions on the range from 0 to 1 (parameters of the beta distribution: α = 1, β = 1) were used for sensitivity and specificity of all three STH infections. For *S. japonicum*, prior specificity mean (SD) for one stool sample was based on our previous work and set to 94.7% (4.0%), and prior sensitivities (SD) for detecting light infection and moderately to heavy infection were set to 54.1% (10.1%) and 75.3% (15%), respectively [33].

The above model was modified to construct three additional models: one model accounted for misclassification error in outcome but not in exposure, one accounted for misclassification error in exposure but not in outcome, and another one did not account for any misclassification error. For models where misclassification error was not accounted for, an individual with any stool sample positive for a particular STH was considered as infection positive for that STH. For *S. japonicum*, epg per participant (intensity of infection) was obtained by averaging the epg of all stool samples collected from a participant, which is the most commonly used method for calculating overall epg per participant [1], [39], [40].

We assumed conditional independence between subsequent tests in our model, meaning in practice that when more than one sample was available from a subject, the test results are independent from each other, conditional on the person's true infection status. In other words, the probability of a positive (or negative) test depends only on the true status, and once this true staus is known, does not depend on any test results from other days. This assumption seemed reasonable, and simplifies the statistical model compared to a model that might account for any between-day dependencies.

WinBUGS software (version 1.4.3, MRC Biostatistics Unit, Cambridge, UK) was used to implement the Gibbs sampler algorithm. Posterior medians of random samples derived from marginal posterior densities were used as point estimates, reported with 95% Bayesian credible intervals (BCI). The programs written in WinBUGS are available upon request to the authors.

## Results

Of the 5624 individuals who agreed to participate in the study at baseline, 2276 (40.5%) constitute the group “at-risk”. The “at-risk” group and those who were not treated with praziquantel or did not provided any stool sample during the follow-up (“not at-risk” group) are compared in Table 1. A higher proportion of people in the “at-risk” group had a positive schistosomiasis test at baseline (23.5%) as compared to those in the “not at-risk” group (10.5%). Because of this discrepancy, there were more rice farmers in the “at-risk” group than in the ‘not at-risk’ group (50.2% vs. 40.9%), since rice farming is associated with *S. japonicum* infection. Having been positive at baseline, however, did not have an impact on the probability of providing a stool sample at follow-up among those people who did receive treatment (75.9% vs. 76.1%).

**Table 1:** Characteristics of the individuals in the “at-risk” and “not at-risk” groups.

Characteristic	At-risk group, no. (%)	Not at-risk group, no. (%)
**N**	2276 (40.5)	3348 (59.5)
**Age (years)**		
<10	658 (28.9)	1193 (35.6)
11–16	399 (17.5)	458 (13.7)
17–40	618 (27.2)	1047 (31.3)
>40	601 (26.4)	650 (19.4)
**Male**	1274 (56.0)	1692 (50.5)
**Rice farming**	1142 (50.2)	1368 (40.9)
**Positive schistosomiasis test at baseline**	534 (23.5)	350 (10.5)

Data collected on 5624 people living in 50 villages of Samar Province, the Philippines, 2003–2004. Individuals in the “at-risk” group were included in the analysis. Individuals in the “not at-risk” group provided stool sample at baseline but were not included in the analysis.

**Figure 1:**
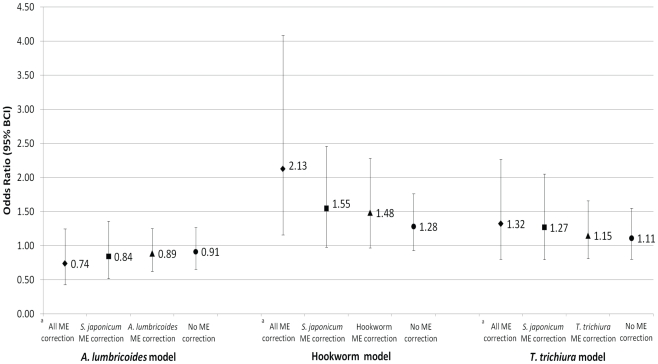
Odds ratio estimates for the exposure variable with and without correction for misclassification error. Exposure variable is respective soil-transmitted helminth infection; All odds ratio estimates are adjusted for age, sex, and occupation; BCI: Bayesian credible intervals; ME: misclassification error; ^a^ Correction of misclassification error in exposure (respective STH infection) and outcome (*S. japonicum* infection) assessment.

Figure 1 displays the OR estimates for the exposure variable (STH infection) from models with and without correction for misclassification error. The OR estimates (95% BCI) for hookworm infection changed from 1.28 (0.93, 1.76) without any adjustment for misclassification error to 2.13 (1.16, 4.08) when both exposure (hookworm infection) and outcome (*S. japonicum* infection) were corrected for misclassification error. For *A. lumbricoides* and *T. trichiura*, the OR changed from 0.91 (0.66, 1.26) to 0.74 (0.43, 1.25) and 1.11 (0.80, 1.55) to 1.32 (0.80, 2.27), respectively. Correction for misclassification error in either exposure or outcome gave intermediate estimates. However, only adjusting for misclassification error in *S. japonicum* had a larger impact on the OR estimates and their 95% BCI than only adjusting for the misclassification error in the STH. In general, misclassification error-adjusted estimates were further away from the null value and had wider confidence intervals than non-adjusted estimates. In addition, the impact of adjusting for misclassification error on OR estimates and their 95% BCI was larger for hookworm which had the lowest sensitivity and specificity values.

Table 2 provides OR estimates for covariates from respective STH models, with and without adjustment for misclassification error. For all three STH models, misclassification error-unadjusted OR estimate for >40 year-old individuals (reference: ≤10 years) was approximately 1.5 times that found in the exposure and outcome misclassification-adjusted model. Also, for all three STH models, OR estimates for males (reference: females) from the misclassification error-adjusted model were considerably different from OR estimates found in the unadjusted model. In general, both exposure and outcome misclassification error-adjusted ORs, and only outcome-adjusted ORs were similar whereas misclassification error-unadjusted ORs and only exposure-adjusted ORs were similar. The estimated 95% BCI from models adjusting for misclassification error in the outcome variables, with or without adjustment for misclassification error in the STH, were wider than those from models without adjustment of the outcome variable. Adjusting for misclassification error of STH only did not impact the width of the 95% BCI of the ORs of other variables in the model.

**Table 2:** Odds ratio estimates for covariates from *A. lumbricoides*, Hookworm, and *T. trichiura* models.

	All ME correction^a^		Outcome ME correction		Exposure ME correction		No ME correction	
Covariates	OR	95% BCI	OR	95% BCI	OR	95% BCI	OR	95% BCI
***A. lumbricoides*** ** model**								
**Age**	Reference: ≤10 yrs							
11–16 yrs	1.05	0.51, 2.11	1.05	0.50, 2.08	1.03	0.62, 1.67	1.04	0.63, 1.68
17–40 yrs	0.49	0.22, 1.07	0.50	0.22, 1.04	0.60	0.34, 1.01	0.61	0.35, 1.03
>40 yrs	0.21	0.09, 0.49	0.22	0.09, 0.49	0.36	0.20, 0.63	0.37	0.20, 0.65
**Sex**	Reference: female							
Male	2.33	1.50, 3.75	2.33	1.48, 3.70	1.83	1.34, 2.50	1.84	1.35, 2.52
**Occupation**	Reference: rice farming							
Non-rice farming	0.42	0.20, 0.86	0.42	0.20, 0.82	0.48	0.29, 0.78	0.49	0.30, 0.79
**Hookworm model**								
**Age**	Reference: ≤10 yrs							
11–16 yrs	1.03	0.49, 2.07	1.07	0.52, 2.12	1.02	0.61, 1.66	1.03	0.62, 1.67
17–40 yrs	0.44	0.19, 0.94	0.48	0.22, 1.00	0.56	0.32, 0.96	0.58	0.33, 1.01
>40 yrs	0.20	0.08, 0.45	0.21	0.09, 0.47	0.34	0.18, 0.60	0.35	0.19, 0.63
**Sex**	Reference: female							
Male	2.12	1.35, 3.34	2.15	1.38, 3.48	1.72	1.26, 2.37	1.75	1.28, 2.40
**Occupation**	Reference: rice farming							
Non-rice farming	0.44	0.21, 0.87	0.45	0.22, 0.88	0.49	0.30, 0.79	0.50	0.30, 0.82
***T. trichiura*** ** model**								
**Age**	Reference: ≤10 yrs							
11–16 yrs	1.09	0.52, 2.16	1.08	0.52, 2.14	1.03	0.62, 1.68	1.03	0.63, 1.68
17–40 yrs	0.54	0.25, 1.15	0.54	0.24, 1.13	0.63	0.36, 1.07	0.62	0.36, 1.05
>40 yrs	0.24	0.10, 0.53	0.24	0.10, 0.53	0.37	0.21, 0.66	0.37	0.21, 0.66
**Sex**	Reference: female							
Male	2.30	1.48, 3.68	2.31	1.48, 3.66	1.82	1.34, 2.50	1.83	1.34, 2.50
**Occupation**	Reference: rice farming							
Non-rice farming	0.43	0.21, 0.85	0.43	0.20, 0.84	0.49	0.30, 0.79	0.49	0.30, 0.79

Odds ratio estimates were calculated using data collected on 2276 people living in 50 villages of Samar Province, the Philippines, 2003–2004; BCI: Bayesian credible intervals; ME: misclassification error; OR: odds ratio;

a Correction of misclassification error in exposure (respective STH infection) and outcome (*S. japonicum* infection) assessment.

## Discussion

To our knowledge, this is the first longitudinal study to estimate the effect of STH infection on the 12-month risk of *S. japonicum* infection in a population where both of these infections are endemic. In addition, this study minimizes several potential biases by including adjustment for misclassification error in both dependent and independent variables, varying sensitivity and specificity of both tests depending on the numbers of samples available, accounting for clustering between individuals within villages, and taking care of other possible confounders. The adjusted model suggests that hookworm infection is associated with increased 12-month risk of *S. japonicum* infection following treatment with praziquantel. The two other STH studied did not have an important effect on the risk of infection with schistosomiasis.

Although our analysis included only about one third of the baseline participants from 50 villages, the longitudinal sample size was large enough for this analysis. When comparing individuals included in and excluded from the analysis, we found more rice farmers in the ‘at-risk’ group than in the ‘not at-risk’ group. This is because more males were treated than females (56.4% vs. 43.6%), and because more rice farmers were infected with *S. japonicum* at baseline. A larger proportion of individuals infected with *S. japonicum* at baseline received treatment [36]. However, this did not have an impact on the probability of providing a stool sample at follow-up among those people who did receive treatment. So, the use of the “at-risk” group of participants is unlikely to introduce selection bias and to affect the validity of our estimates.

Our results show that OR estimates for all three STHs are pulled away from the null value when the OR estimates are adjusted for misclassification error. This effect of non-differential misclassification has long been recognized, although this is not always the case when exposure and outcome variables are dependent, a discrete variable assumes more than two values, or there is misclassification error in the confounding variable [21], [23], [41], [42].

The effect of misclassification on the OR estimates of the association between STH and the risk of *S. japonicum* infection differed for the three STHs under study. The magnitude of impact of misclassification error depends on the sensitivity, specificity, and true prevalence of the variable(s) of interest. The relative change in the OR estimates between the unadjusted model and the model adjusting for misclassification error of STH and *S. japonicum* was larger for hookworm than the other STHs. This is likely to be due to the considerably lower sensitivity (single stool sample) of the Kato-Katz for hookworm as compared to that for *A. lumbricoides* and *T. trichiura* [43].

Two studies have reported estimates of cross-sectional association between hookworm infection and infection by another schistosome species (*S. mansoni*). Keiser and others (2002) reported an OR of 2.25 (95% CI: 1.31, 3.85) from their study conducted among 325 school children in Côte d'Ivoire [2]. Fleming and others (2006) reported an OR of 2.95 (95% CI: 2.19, 3.98) from a study conducted among 1332 individuals in Brazil [17]. Their results, which did not adjust for misclassification error, could be due to the cross-sectional nature of their study, which could increase the association between the prevalences of hookworm and schistomiasis. It is also possible that the association between hookworm and schistosomiasis is larger for *S. mansoni* than for *S. japonicum* or that the Kato-Katz performs better for the diagnosis of *S. mansoni*, thus reducing the effect of misclassifaction error. Moreover, temporality of the association could not be ascertained because of the cross-sectional design of these studies. Longitudinal design of our study allowed us to assess the impact of hookworm infection on the incidence of schistosomiasis japonica, after adequate adjustment for misclassification error. Even though the OR may overestimate somewhat the relative risk, these measures are likely to be reasonably close in our study since the risk of re-infection was in the order of 13%.

Important changes in OR estimates for other covariates were also observed. The OR estimates for covariates when only *S. japonicum* data (outcome) were adjusted for misclassification error were very close to the OR when both *S. japonicum* and STH data were adjusted. In contrast, the OR estimates for covariates when only STH data (exposure) were adjusted for misclassification error were very close to unadjusted OR estimates. This is because the strength of the association between the covariates and *S. japonicum* infection was considerably larger than the confounding effect of STH infections. Nevertheless, even correction for misclassification error in the outcome variable only was capable of changing estimate of effect of some of the covariates on the risk of *S. japonicum* infection. This has important implications for the assessment of the confounding effect of these variables and their association with the risk of *S. japonicum* infection. We also observed wider confidence intervals for all misclassification error-adjusted ORs. This results directly from incorporating uncertainty in estimating infection status [21], [44].

The largest impact of misclassification error was observed for the association between hookworm and *S. japonicum*, which was negligible in the unadjusted model and important on the adjusted one. Several authors have provided numerical examples in their publications showing larger effects of joint misclassification of both exposure and outcome [22], [41], [45]. For *A. lumbricoides* and *T. trichiura*, OR point estimates indicate a negative and a positive relationship, respectively, but of a smaller magnitude.

The efficacy of praziquantel for the treatment of schistosomiasis has been reported to range between 71% and 99% in published literature [46], [47], [48]. However, more recent papers have reported an efficacy of praziquantel for the treatment of schistosomiasis around 96% [46], [47]. The “at-risk” group size is likely to be affected by a lower efficacy as treatment with praziquantel does not completely cure everyone who has the infection. In our study, we assumed 100% efficacy of praziquantel for the treatment of schistosomiasis and decided not to adjust for a lower efficacy of praziquantal. This would have required yet another level of uncertainty for only a small proportion of the population (the efficacy is very high), and is unlikely to have changed our conclusions. Another limitation of this study is that our model assumes conditional independence of test results within each individual given the latent true infection status which is always uncertain. To assess conditional dependence we first have to build a more complex model assuming that there is at least some dependence. This allows examination of the size of the dependence parameter and whether or not its use is meaningful [49]. Exploring such a complex model is beyond the scope of this paper. However, several authors have noted that overlooking conditional dependence does not substantially change parameter estimates [49]–[51]. Our results were adjusted for risk factors most often reported to be associated with schistosomiasis, and often shared with hookworm, such as age, gender, occupation, and the village where people live. Although some additional unmeasured confounding factors may explain the observed association, such factors would need to have a very strong relationship with both hookworm and schistosomiasis to modify our conclusion.

Our data suggest that hookworm infection is associated with increased 12-month cumulative incidence of *S. japonicum* infection. Such important associations might be missed in analyses which do not adjust for misclassification errors. Our findings have important implications for control of these infections in regions where these worms are co-endemic. Effective control of one helminth can lead to reduction in incidence of another and help to reduce the overall burden of helminthic infection in affected regions.

## Supporting Information

Checklist S1 STROBE Checklist.
(0.09 MB DOC)
